# Women's participation in mobile phone surveys in Mozambique: Findings from a qualitative study

**DOI:** 10.1111/tmi.70006

**Published:** 2025-07-19

**Authors:** Rosemary Morgan, Yolanda Manganhe, Celso Monjane, Milly Nakatabira, Helen Kuo, Cremildo Manhiça, Ferão Mandlate, Milton Sengo, Midalia Uamba, Almamy Malick Kante, Ivalda Macicame, Agbessi Amouzou

**Affiliations:** ^1^ Department of International Health Johns Hopkins Bloomberg School of Public Health Baltimore Maryland USA; ^2^ Instituto Nacional de Saude Maputo Mozambique

**Keywords:** gender, mobile phone surveys, women's participation

## Abstract

**Objectives:**

The use of mobile phone surveys in low‐ and middle‐income countries is increasing as a low‐cost and rapid alternative to in‐person interviews. However, ensuring they are representative of women and, when women are included reducing potential response bias and harm are important considerations. To improve women's participation in phone surveys, we conducted a qualitative study in Mozambique to better understand women's experiences of participating in mobile phone surveys.

**Methods:**

This study was part of the Rapid Mortality Mobile Phone Survey (RaMMPS) project implemented in Mozambique to test the use of mobile phone interviews for childhood mortality measurement at the national level. We conducted a qualitative study with 32 women who had previously participated in the RAMMPS mobile phone survey. Interviews were conducted both in‐person and over the phone. Thematic analysis was done manually using the Framework approach.

**Results:**

Gender‐related considerations that emerged from the data regarding women's participation included women's access to mobile phones, the reduced time burden and convenience of participating in mobile phone interviews compared to in‐person interviews, difficulties ensuring privacy in mobile phone surveys, the effect of the interviewer's gender on participant responses, and women's safety concerns.

**Conclusion:**

Important considerations for including women in mobile phone surveys relate to efforts to reduce response bias and mitigate harm, such as ensuring privacy and considering the gender of the data collector. Addressing these issues is crucial to improving women's participation and experience in mobile phone surveys.

## INTRODUCTION

The use of mobile phone surveys to collect data in low‐ and middle‐income countries (LMICs) is increasing [1, 2]. This was particularly true during the COVID‐19 pandemic when in‐person interviews were suspended despite an urgent need to collect timely and relevant population health data [3, 4, 5]. Compared to traditional face‐to‐face interviews, phone surveys are also often viewed as a low‐cost and rapid alternative, making them appealing to many researchers [6, 7, 8, 9].

How representative phone surveys are, however, remains an important consideration [10, 11, 12], particularly their ability to reach women [5, 6]. Women's inclusion in mobile phone surveys depends a lot on their access to mobile phones. The gender gap in phone ownership in LMICs is well established [13, 14]. While mobile ownership among women in LMICs is increasing, with approximately 83% of women in LMICs owning a mobile phone, 60% owning a smartphone, and 66% using mobile internet, women are still less likely to own a mobile phone (particularly a smartphone) than men [13]. This is particularly true for marginalised and underserved women, including women who live in rural areas, have low literacy, less access to financial resources, or have a disability [13]. While recent years have seen the gender gap in smartphone ownership between women and men narrow (60% vs. 69%, respectively), it translates into approximately 200 million fewer women than men who own a smartphone [13] in LMICs.

Difficulties in accessing women in mobile phone surveys, especially rural and lower‐educated women, have been reported in other studies. Selection biases were found in national phone surveys during the COVID‐19 pandemic in Ethiopia, Malawi, Nigeria, and Uganda, with respondents more likely to be heads of households, older, and better educated [11]. Challenges in reaching women due to connectivity issues and lack of access to mobile phones or airtime were also reported in India [15]. A study in Ghana found substantial coverage bias due to fewer women, rural, and older people completing phone surveys compared to traditional household surveys [16]. In rural India, gaps in phone access due to male management of household phones limited women's representation in phone surveys [6]. Within the India study, women reported lower daily phone use compared to men, with one‐third using the phone only in emergencies [6]. The implications of women's lack of representation in phone surveys are severe, as their lack of inclusion further exacerbates the global gender data gap—the absence of data on and about women's lives in official statistics—which in turn affects decision‐making and priority‐setting on women's health, wellbeing, and livelihoods [6].

In addition to considerations around access and use of mobile phones, which can impact women's participation in mobile phone surveys, there are important gender‐related considerations that researchers need to take into account regarding women's participation. These include women's safety, issues related to privacy and confidentiality, cultural and gender norms regarding the types of questions asked and by whom, and the time burden that mobile phone survey participation might inflict upon women [6, 7, 15]. These issues are important to consider so that women's participation in mobile phone surveys does not lead to unintended negative consequences, such as exposing them to violence within the home, increasing women's discomfort, or affecting the validity and reliability of the responses provided, for example by women refusing to answer or answering falsely due to others being present or the presence of a male enumerator [6, 7, 15].

In order to better understand women's experiences of participating in mobile phone surveys, we conducted a qualitative study in Mozambique with women who had previously participated in a mobile phone survey. Mozambique is one of the world's poorest countries [17]. In 2022, Mozambique scored 0.461 on the Human Development Index (which takes into account gender equality), ranking 183 out of 193 countries [18]. While the labor force participation rate of men and women is relatively the same (79.8% and 78.2%, respectively) [19], women are less represented in non‐subsistence sector employment [20]. Women's adult literacy rate (percentage of people aged 15 and over) is much lower compared to men's (49% and 72%, respectively) [19]. In addition, the percentage of women who reported having an account at a financial institution in 2021 is much lower than men (38.7% and 61%, respectively) [19], while in 2023, 51.2% of women reported participating in major household decisions [19]. The rate of female phone use in Mozambique is also significantly less than that for men. According to Global System for Mobile Communications (GSMA) data in 2021, approximately 64% of men owned a mobile phone, compared to 47% of women [21].

## METHODS

This study was part of the Rapid Mortality Mobile Phone Survey (RaMMPS) project implemented in Mozambique to test the use of mobile phone interviews for childhood mortality measurement at a national level [22].

### Study design

We conducted a qualitative study with women who had previously participated in the RAMMPS mobile phone survey. RaMMPS used an existing national database of phone numbers collected as part of an existing national mortality surveillance system called the Countrywide Mortality Surveillance for Action (COMSA) [22]. Between June and November 2023, trained data collectors called 45,000 phone numbers to reach women of reproductive age, aged 15‐49 years. The final sample size for the RaMMPS survey was 13,235 women.

Participants in the qualitative study were selected through de‐identified records from the RaMMPS study. In order to be eligible to participate, respondents had to be between the ages of 18–49, had previously participated in the RaMMPS survey, and lived in the Southern Maputo region. Initial contact was made via mobile phones to confirm their willingness to participate. Semi‐structured interviews were conducted both in‐person and over the phone. Mobile phone interviews allowed for real‐time data collection, while in‐person interviews provided observational data on privacy and safety and enabled in‐depth exploration of topics. Conducting both types of interviews helped explore the impact of interview modality on the quality of information collected. The choice of mobile phone or an in‐person interview depended on participants' location due to budget and travel feasibility. There was a 24‐hour window between the initial call and the interview to allow participants to consider or withdraw their participation. A total of 32 interviews were conducted, split evenly between mobile phone (x16) and in‐person (x16) interviews. Participants were from both rural (x16) and urban (x16) areas. Each area had eight mobile phone and eight in‐person interviews. The number of interviews was based on previous qualitative research experience and expected saturation.

### Data collection

Interviews were conducted in December 2023 by trained INS staff in Portuguese. They were held at convenient times and locations for participants. For the in‐person interviews, interviews were conducted at the place and time of the participant's choosing. Only one interviewer was present for each interview and all interviews were conducted by female data collectors. All interviews were digitally recorded with permission; otherwise, handwritten notes were taken. On average, each mobile phone interview lasted about 15–30 min, while in‐person interviews lasted about 30–45 min. After each interview, the interviewer wrote their reflections in a fieldwork memo, indicating whether the interview was conducted in private or any issues which may have emerged during the interview. Digital files were stored on password‐protected computers accessible only to the study team. Recordings were transcribed verbatim and translated into English.

### Data analysis

Thematic analysis was done manually by two researchers using the Framework approach [23]. The Framework approach has five distinct stages: transcription, familiarisation with interviews, coding, mapping/charting of data in a matrix, and interpretation of the data [24]. The Framework approach has been successfully applied across qualitative health research and helps to build transparency and validity of the research through providing a systematic and documented approach to analysis [24]. During the coding phase, a codebook with *a priori* and emergent codes was developed through team discussions. Codes were systematically applied to all transcripts and fieldwork memos. The coded text was then inputted into matrices, which allowed us to identify patterns and develop interpretations through the overall synthesis of results.

### Ethical clearance

The National Bioethics Committee for Health in Mozambique (reference 98/CNBS/2023) and the Johns Hopkins Bloomberg School of Public Health (reference IRB00021536) approved the study protocol. Respondents were given the opportunity to discuss their participation with their family and friends and then consent later. Before the interview, participants were informed about the study objectives, risks, benefits, and their right to refuse participation, and informed consent was obtained.

## RESULTS

This study explored women's participation in mobile phone surveys in order to better understand factors affecting their participation and outcomes of their participation. This included factors which could introduce bias in women's responses and the time burden of participating in mobile phone interviews compared to in‐person interviews. We also explored how women's participation may lead to unintended negative consequences, potentially affecting their wellbeing and safety. While some of the issues identified are important considerations for both men and women participating in mobile phone surveys, some have different implications for women due to existing gendered norms, roles, and relations.

Key themes relevant to women's participation in mobile phone surveys which emerged from the study are included below. The section starts by discussing findings related to women's access to mobile phones as reported by participants. This is followed by important gender‐related considerations which emerged from the data regarding women's participation. These included: the reduced time burden and convenience of participating in mobile phone interviews compared to in‐person interviews, difficulties ensuring privacy in mobile phone surveys, the effect of the interviewer's gender on participant responses, and women's safety concerns.

### Women's access to mobile phones

The majority of respondents in the study reported owning their mobile phones and that access to a mobile phone was not an issue. A few respondents, however, stated that they borrowed their phone from their husband or children.

One respondent who said that her husband owned the phone reported that she found it challenging to access it, as when he was unavailable she did not have access to it, despite being the one to put credit on it.

“*It's very difficult, he has to come back so I can borrow his phone… now, I'm the one who usually recharges it… yes, yes, yes. Yes, when I want to talk to people far away I have to wait for him to get back from work to call me so I can talk*.” (004‐Mobile phone interviewee)

### Reduced time burden and convenience

The time burden placed on participants is an important consideration in research. This is particularly relevant for women who often have less free time compared to men due to their household and caregiving responsibilities, often coupled with work outside the home. Overall, the mobile phone interviews in this study were less of a time burden for participants compared to in‐person interviews, averaging 15–30 min compared to 30–45 min, respectively.

Many respondents also preferred mobile phone interviews to in‐person interviews as they found them to be more practical, flexible, and convenient, often allowing them to multitask while participating in the interview.

“*[…] In case I want to go out, then if you tell me that we should talk, I may feel uncomfortable sitting down, but on the phone, we can talk while I also go about my business as we speak*.” (002‐mobile phone interviewee)

“*The cell phone reduces a lot of things, you can call and talk, sister, we are coming to the house to talk, it can be difficult, but on the phone we can talk*.” (002‐mobile phone interviewee)

“*You can call me if you are far from the city, say tomorrow we will have an interview I will not be able to participate, I think it is more practical on the phone*.” (0009‐in‐person interviewee)

### Ensuring privacy

Ensuring privacy is an important consideration within all research, whether conducted over the phone or in‐person. This is particularly important for women whose privacy is inherently linked with their safety. While many respondents stated that they would have no problem finding a private place for a mobile phone interview, some respondents said outright that it would be difficult for them to find a private place to participate in a mobile phone interview.

“*I can say it's not easy (to find a private place) because there are times when there are a lot of people here, you can't sit down and talk but if you had time like now that I'm alone there's no problem*.” (0003‐mobile phone interviewee)

Participants who were near other people during a phone interview and worried about being overheard stated that they may not answer a question directly or would be unable to answer.

“*I can't answer where others are listening. Yes, I would answer differently*.” (0002‐mobile phone interviewee)

“*I can answer with reservations because there are things that I can't answer when I'm with someone else*.” (00010‐in‐person interviewee)

Ensuring privacy was particularly important when the interview included sensitive or personal questions. One participant reported that she would be uncomfortable sharing information on sensitive issues such as family planning or intimate aspects of health, especially when there were men present.

“*If to ask questions, for example wanting to talk about issues related to women's intimate health, I[…] if we were where men are and not women, perhaps I wouldn't be comfortable, because since you would be talking about issues related to women while men are involved, if it were just women and I would respond without reservation*.” (0006‐in‐person interviewee)

It became clear from responses and fieldwork memos that researchers have less control over ensuring privacy and confidentiality during mobile phone interviews compared to in‐person interviews. During some mobile phone interviews, for example, participants talked to others in the room which indicated that they were not in a private location or were distracted during the interview (although this was not always easy to determine). During in‐person interviews, interviewers were able to pause the interview if someone entered the room or came near and start it back up again once privacy was ensured.

### Effect of interviewer's gender on participant responses

The gender of the person conducting the interview is an important consideration, particularly if a study involves sensitive information. Female respondents may be less inclined to respond to specific questions if the interviewer is male or may even answer a question untruthfully if they are embarrassed, which can affect the validity of a study's findings. Cultural norms, for example, may prevent women from sharing sensitive information related to their sexuality or reproductive health with men, or may even prevent women from being allowed to speak to men who are not within their immediate family. When asked if they would prefer to talk to a female or male interviewer, most preferred to be interviewed by female interviewers, particularly if the interview involved sensitive information.

“*It's better if it's a woman because she and I will understand each other*.” (001‐mobile phone interviewee)

“*The woman is easy to talk to, explain. But man, but if that's the case, there's no way I could answer you*.” (0003‐mobile phone interviewee)

“*When he asks things like that [i.e., sensitive topics] I can get scared, hey why are you asking me that, but when a woman asks anything I have to respond in a good way because she is a woman*.” (0004‐mobile phone interviewee)

Many women stated that they would open up more to a female interviewer compared to a male interviewer or would answer questions differently.

“*It would be different when you are talking to another woman. Yes, it's different because you open up when you're a woman, but when you're a man, you're not used to it*.” (0005‐mobile phone interviewee)

“*[I] would answer differently because men I can't answer everything they're asking*.” (0006‐in‐person interviewee)

“*[A male interviewer] affects […] the way I can answer questions*.” (0009‐in‐person interviewee)

### Effect of interviewer's gender on participant safety

Safety is an important consideration for women, whether in their daily lives or when participating in research. Within this study, participants stated safety concerns when participating in mobile phone surveys when the interviewer was male. These included concerns over being scammed and concerns over how their husband might respond should they find out they were talking to another man.

Some respondents worried that an interview over the phone was not legitimate, and the interviewer was trying to get personal information out of them. This predominately came up among in‐person respondents as opposed to the mobile phone respondents, which was likely due to the nature of the interview and the fact that the mobile phone interviews used protocol to establish creditability at the start of the interview.

“*Sometimes some questions can make you uncomfortable on the phone due to doubts. Dealing with someone you can't see, you may have doubts, especially now that the world is full of very bad things. You don't know who you're dealing with; the person may ask, ‘Where do you live? Do you have a bank account? Do you have M‐pesa? Do you have e‐mola?’ You don't know who you're dealing with*.” (008‐in‐person interviewee)

“*It's strange, when someone you don't know calls you and starts asking questions, sometimes you're… a lot of things pop into your head, could this person be serious? Maybe this person isn't… he really wants to ask me about my life, what he wants to do with my life, there are always a lot of questions*.” (0005‐mobile phone interviewee)

“*Yes (might be comfortable participating in a phone interview), but with doubt, not knowing if they are scammers. I prefer in‐person*.” (006‐in‐person interviewee)

One woman was concerned about her husband's reaction when she received a call from a man. She explained that her husband might think she was cheating on him, emphasising that she only wanted calls from women.

“*It must be a woman… well… if it's a man, my husband might think it's a lover*.” (003‐mobile phone interviewee)

While this concern only came up with one respondent, we felt it was an important data point to highlight given the seriousness of the concern and the potential for negative consequences.

## DISCUSSION

Our study identified a number of important gender considerations related to women's participation in mobile phone surveys. Within our study, many respondents preferred mobile phone interviews compared to in‐person interviews due to their flexibility and reduced time burden. Qualitative phone interviews in Nigeria also found that women viewed mobile phone surveys as convenient and flexible [7]. The potential to reduce women's time burden is an important gender consideration given women's increased household and caregiving responsibilities [25, 26]. While the reduced time burden may be partly due to the flexibility and convenience that mobile phone interviews allow, it may also be due to the limited rapport that interviewers are able to foster over the phone compared to in person. In‐person interviews offer an opportunity for researchers to build rapport and trust during the interview, allowing them to probe for more detailed responses. A study in Uganda, for example, found that women saw mobile phone surveys as being convenient but missed the close interaction of in‐person surveys [27]. For interviews that deal with more sensitive topics, building rapport might be particularly important, and mobile phone surveys may not be the best modality to collect this information.

Ensuring privacy and confidentiality during mobile phone interviews is an important gender consideration, especially when interviews include sensitive information or personal questions. Within our study, participants worried about being overheard, which they stated would impact their responses. Many participants preferred female interviewers when sensitive topics were discussed, stating that they were likely to respond differently or not at all to male interviewers. Challenges in obtaining reliable and quality information from women in mobile phone surveys were also reported in India [15]. Privacy issues and response bias were noted when women answered phones on speaker or in the presence of others [15]. Another study in India found that women's refusal rate to participate was higher for male enumerators compared to female enumerators [6].

Safety is another important gender consideration, which in our study was related to the gender of the data collector. Some participants equated male callers with the potential of being scammed. One participant preferred female interviewers to avoid the potential of being accused of infidelity by their male partner. Findings from a mHealth intervention implemented through a randomised control trial in Bangladesh which used interactive voice messages to promote contraceptive use after menstrual regulation found that self‐reported experiences of intimate partner violence increased among the intervention group compared to the control group [28]. The authors discussed possible reasons for this increase. As participants received the messages while they were at home, they theorised that this contributed to suspicions of infidelity as well as the content potentially being viewed as unacceptable when the message was overheard [28]. While the Bangladesh study did not employ a mobile phone survey, the modality to relay information (i.e., a mobile phone) was the same, potentially leading to similar outcomes.

Women's inclusion in mobile phone surveys is dependent on their access to mobile phones. Mobile phone surveys conducted in Ethiopia, Malawi, Nigeria, Uganda, India, and Ghana have reported difficulties in accessing women due to selection biases [6, 11, 15, 16]. Women who lived in rural areas, were lower‐educated, or younger were less represented within many of the surveys. In some instances, men's control over the household mobile phone limited women's participation [6]. Mobile phone surveys risk excluding women's voices, particularly women who are more vulnerable due to their intersecting identities. Women's exclusion from research (whether collected through mobile phones or more traditional methods) can further exacerbate the global gender data gap, where information about women's health, wellbeing, and livelihoods is made invisible [6, 29]. The global gender data gap can further be exacerbated by the exclusion of gender‐specific questions even when women are included [29, 30, 31]. Participation in research can also negatively affect women's safety and as a result, researchers need to ensure that any potential emotional or physical harm is mitigated. In the section below, we include recommendations for improving women's participation and experience in mobile phone surveys.

## IMPROVING WOMEN'S PARTICIPATION IN AND EXPERIENCE OF MOBILE PHONE SURVEYS

### Women's representation within mobile phone surveys

Many mobile phone surveys rely on random samples. Random sampling in survey design, however, can limit accessibility, particularly for more vulnerable groups such as women. In order to increase the representativeness of women in mobile phone surveys, a mixed mode survey design, which uses two or more methods to collect data or sample participants, may be needed. In addition to random sampling, purposive or snowball sampling can be employed. This can include referrals and/or scheduling appointments and callbacks. Referrals involve asking the person who answered the phone to refer an eligible woman in the household. This process has been found to improve the representation of women, especially those from vulnerable groups (e.g., young women, poorer women, and those living in areas with low connectivity), but at a higher financial cost [5]. Scheduling appointments and callbacks has also been found to increase the representation of women by mitigating low phone passing rates (which referrals on their own may not solve) [6]. This method, however, requires frequent callbacks to reach a woman, making it more labour‐intensive. While the use of purposive or snowball sampling on its own can lead to an unrepresentative or over‐representative sample, combining these approaches with random sampling can help to ensure generalisability while increasing accessibility for vulnerable groups, such as women.

### Privacy, response bias, and safety

Ensuring privacy during mobile phone interviews is challenging as the interviewer has little control over the interview location. Ensuring privacy can reduce response bias, improve data reliability, and mitigate harm. This includes conducting the interview in a private location with no one else present and not using the speaker option. Interviewers should note if the respondent is alone or using the speaker phone and adjust questions accordingly. This means that certain personal questions or modules, such as those about violence, sexual health, or intra‐household conflict, may need to be excluded from the interview [15]. Respondents should be informed that they can stop the interview at any time and a code word can be used to indicate the interview is no longer private.

Using female enumerators can reduce response bias and mitigate harm, especially with sensitive questions. This can address gender norms and women's hesitancy to discuss topics like sexual and reproductive health, childbirth, breastfeeding, menstruation, and domestic violence with men [6]. These topics can be deeply personal and/or culturally sensitive, and women may be hesitant to talk about such topics with men, or it may be inappropriate for them to do so. Female enumerators can help build trust and rapport in phone interviews, allowing respondents to speak more freely. In some contexts, it may be difficult or more labor‐intensive to hire female data collectors due to their lack of availability or experience. Hersh et al. [6] hired an all‐female survey team for a COVID‐19 survey in India and reported facing operational challenges due to female enumerators being less experienced and qualified compared to the predominantly male enumerator applicants. Consequently, more time and resources were needed for training the female data collectors. As women are increasingly hired as enumerators, however, this will become less of an issue. As is common practice, mobile phone interviews should have clear protocols in place to provide information about the study up front in order to establish the credibility of the interviewer and mitigate respondent's worries over being scammed.

## LIMITATIONS

There are several limitations to this study. An important limitation of trying to understand mobile phone ownership among women through a mobile phone survey is that women who do not have access to a mobile phone will automatically be excluded. Using mobile phone surveys to understand mobile phone ownership is, therefore, unlikely to provide a complete picture. Our sample drew from women who participated in the initial RaMMPS survey, as we wanted to ensure that participants had previously participated in a mobile phone survey, which meant that women who did not have access to, or had very limited access to, a mobile phone were excluded. These same limitations will be present in mobile phone surveys in general, which will be restricted to individuals who have access to a mobile phone, excluding a proportion of the population.

Respondent's location influenced the type of interview they were given—whether in‐person or over the phone—which may have impacted the results by type of respondent. Respondents tended to favour the modality of interview in which they were participating. Respondents who were participating in in‐person interviews tended to prefer in‐person interviews, while respondents participating in mobile phone interviews tended to prefer mobile interviews. This could imply bias in terms of the interviewee stating what they thought the interviewer wanted to hear.

We also did not record who picked up the phone and, when a male member of the household picked up, whether they were able to pass the phone to a female family member. In addition, for interviews conducted over the phone, it was difficult to determine whether the respondent conducted the interview while on speaker phone or whether there were other people present. In some instances, participants talked to others in the room which indicated that they were not in a private location; however, this was not always easy to determine. We were, therefore, unable to determine the extent to which lack of privacy may have impacted response bias of the mobile phone interviews.

## CONCLUSION

Many women find mobile phone surveys more convenient and flexible than in‐person interviews, as they are less time‐consuming and allow for multitasking or scheduling at a convenient time. However, gender‐related issues such as maintaining privacy, response bias, and respondent safety, especially with male enumerators, need to be addressed. More research is needed to explore how participation in mobile phone interviews may lead to unintended negative consequences for women. Failing to consider and address these issues can reduce data reliability and negatively impact female participants. Addressing these issues is crucial to improving women's participation and experience in mobile phone surveys.

## FUNDING INFORMATION

This study was made possible with financial support from the Gates Foundation (INV‐023211). The funder had no role in study design, data collection, data analysis, data interpretation, or writing of the report.

## CONFLICT OF INTEREST STATEMENT

The authors declare no conflicts of interest.
